# *Plasmodium vivax* Tryptophan Rich Antigen PvTRAg36.6 Interacts with PvETRAMP and PvTRAg56.6 Interacts with PvMSP7 during Erythrocytic Stages of the Parasite

**DOI:** 10.1371/journal.pone.0151065

**Published:** 2016-03-08

**Authors:** Kriti Tyagi, Mohammad Enayet Hossain, Vandana Thakur, Praveen Aggarwal, Pawan Malhotra, Asif Mohmmed, Yagya Dutta Sharma

**Affiliations:** 1 Department of Biotechnology, All India Institute of Medical Sciences, New Delhi, India; 2 Malaria group, International Centre for Genetic Engineering and Biotechnology, New Delhi, India; 3 Department of Emergency Medicine, All India Institute of Medical Sciences, New Delhi, India; Ehime University, JAPAN

## Abstract

*Plasmodium vivax* is most wide spread and a neglected malaria parasite. There is a lack of information on parasite biology of this species. Genome of this parasite encodes for the largest number of tryptophan-rich proteins belonging to ‘Pv-fam-a’ family and some of them are potential drug/vaccine targets but their functional role(s) largely remains unexplored. Using bacterial and yeast two hybrid systems, we have identified the interacting partners for two of the *P*. *vivax* tryptophan-rich antigens called PvTRAg36.6 and PvTRAg56.2. The PvTRAg36.6 interacts with early transcribed membrane protein (ETRAMP) of *P*.*vivax*. It is apically localized in merozoites but in early stages it is seen in parasite periphery suggesting its likely involvement in parasitophorous vacuole membrane (PVM) development or maintenance. On the other hand, PvTRAg56.2 interacts with *P*.*vivax* merozoite surface protein7 (PvMSP7) and is localized on merozoite surface. Co-localization of PvTRAg56.2 with PvMSP1 and its molecular interaction with PvMSP7 probably suggest that, PvTRAg56.2 is part of MSP-complex, and might assist or stabilize the protein complex at the merozoite surface. In conclusion, the PvTRAg proteins have different sub cellular localizations and specific associated functions during intra-erythrocytic developmental cycle.

## Introduction

*Plasmodium vivax* is the major cause of malaria outside Africa, mainly affecting Asia and the Americas. Increasing cases of severe disease including cerebral malaria and deaths associated with *P*.*vivax* have challenged the conventional thought that this parasite is capable of causing only non fatal mild disease. Unfortunately, despite large burden of the disease, *P*.*vivax* is neglected due to absence of continuous *in vitro* culture system and the low parasitemias associated with natural infections [1]. Due to technical problems associated with *P*.*vivax*, existing research efforts have largely focused on *P*.*falciparum*; thus, reports of characterized proteins from *P*.*vivax* remain few and functions for many others are speculated on the basis of their counterparts in other species. Moreover, increasing drug resistance has also raised the concern about the future intensification of the *P*.*vivax* malaria. Therefore, better understanding of the parasite pathophysiology and biological mechanisms are required for the containment of the disease.

*Plasmodium* genome codes for many gene families which have been related to several functions such as the host immune evasion, host cell modification and cytoadherence [2–7]. Tryptophan rich proteins of *Plasmodium* have been put forward as potential drug/vaccine targets [8–11] including PypAg 1 and PypAg 3 from murine malaria parasite, *P*.*yoelii* [8] and their orthologues, PfTryThrA [9] and PfMaTrA [12], respectively, in human malaria parasite *P*.*falciparum*. The PArt, another tryptophan rich protein of *P*.*falciparum* was shown to be involved in parasite defense mechanisms against the spiked doses of artesunate [13]. Genome sequence of *P*.*vivax* revealed eight novel gene families of which ‘Pv-fam-a’[2] codes for 36 proteins unusually rich in tryptophan residues. Almost all *P*.*vivax* Tryptophan Rich Antigens (PvTRAgs) are expressed in blood stages, having peak expression during early and schizont stages of the parasite [14]. Previous studies have shown that PvTRAgs can induce the humoral as well as cellular immune responses during *P*.*vivax* infection and most of them exhibit sequence conservation [10, 15]. Some of these PvTRAgs are also known to exhibit human erythrocyte binding activity which may be associated with rosetting or red cell invasion phenomenon [16, 17].

In an attempt to broadly understand the possible biological roles of ‘Pv-fam-a’ family of proteins, two PvTRAgs; PvTRAg36.6 and PvTRAg56.2 were selected here which were predicted to have distinct cellular location using various computational softwares [18–23]. PvTRAg 36.6 (PlasmoDB ID: PVX_112690) was predicted to have apicoplast transit peptide sequence and selected for characterization here since apicoplast serves as an excellent drug target being prokaryotic in its origin. This protein is also among the ten RBC binding PvTRAgs which was presumed to be involved in rosetting [16]. The second protein PvTRAg56.2 (PlasmoDB ID:PVX_088850) was predicted to be nuclear targeted and being syntenic to previously characterized *P*.*falciparum* tryptophan/threonine-rich antigen [24] was also selected for characterization. We have identified here the interacting partners of these two proteins by screening *P*.*vivax* cDNA using bacterial two hybrid system. Further, we have assessed their sub-cellular localization in *P*.*vivax* as well as in heterologous *P*.*falciparum* culture system by GFP trafficking. Together, these results suggest that being distinctly located and interacting with different proteins these PvTRAgs may have diverse roles to play in parasite asexual blood stages.

## Materials and Methods

### Ethics statement

Blood samples were collected from malaria patients infected with *P*.*vivax*. The informed written consent was obtained from the individuals prior to blood collection. Study protocol was approved by the Institute Ethics Committee of All India Institute of Medical Sciences (Approval number IESC/T-428/30.11.2012). All animal experiment protocols were approved by the Institutional Animal Ethics Committee of All India Institute of Medical Sciences (Approval number 494/IAEC/09). The ICMR and GCP guidelines were followed to perform human studies and CPCSEA guidelines were followed to perform experiments on animals.

### *P*.*vivax* cDNA library construction in pTRG vector

Blood samples from *P*.*vivax* infected individuals were passed through CF-11 column to deplete human leukocytes as described earlier [25]. Infected RBCs were then lysed with saponin (0.1%) and parasite pellets obtained after centrifugation at 10,000 x g were washed two times with 1 x PBS. Total RNA was extracted using TRIzol reagent following the method described by kyes et al [26]. The *P*.*vivax* cDNA library was constructed from 5 μg of total RNA using In-Fusion SMARTer™ Directional cDNA Library Construction Kit (Clontech laboratories Inc., Terra Bella Ave., CA USA) following manufacturer’s instructions. The cDNA synthesized with SMARTer ends were ligated to linearlized vector pTRG. Transformants obtained after pTRG-cDNA library transformation in the XL-1 Blue MRF^-^ Kan *E*.*coli* cells were pooled and stored as glycerol stocks.

### Bacterial two hybrid library screening

To identify the putative protein partners, PvTRAg36.6 and PvTRAg56.2 gene fragments encoding exon 2 were amplified using primers 5’- GAATTCC*GGATCC*GAAGCTATGCCCAAATTTC—3’ and 5’- *CTCGAG*CTTTCTAACTTTTTTGACC– 3’ for PvTRAg36.6, and 5’–*GAATTC*CGGATCCTTCTTCAGTAAAAAGTCGAAC– 3’, and 5’–*CTCGAG*CACACTAAGAGCATTTTCTCC—3’ for PvTRAg56.2 (restriction sites are italicized), and cloned in bait pBT plasmid (BacterioMatch® II two-hybrid system, Agilent technologies, Santa Clara, CA, USA) using *BamH1* and *Xho1*cloning sites. The screening experiment consisted of co-transforming recombinant pBT-PvTRAg36.6 or pBT-PvTRAg56.2 with *P*.*vivax* cDNA library. Library screening was carried out as described earlier [27]. Plasmids were isolated from confirmed positive clones and sequenced as described earlier [28]. BLAST search of DNA sequence was done against PlasmoDB (www.plasmodb.org) database to identify the putative interacting proteins.

### Yeast-two hybrid assay

To confirm the interaction between the PvTRAg and its identified putative interacting partner, Matchmaker GAL-4-based yeast two-hybrid system III (Clonetech) was used. Gene fragment encoding exon 2 of PvTRAg36.6 and PvTRAg56.2 was amplified (using primers *5’–CCCGGG*GGAAGCTATGCCCAAATTTC– 3’ and 5’–*GGATCC*CTTTCTAACTTTTTTGACCAC– 3’ for PvTRAg36.6, and 5’–*CCCGGG*GTTCTTCAGTAAAAAGTCGAAC—3’ and 5’- *GGATCC*CACACTAAGAGCATTTTCTCC– 3’ for PvTRAg56.2, restriction sites are italicized) and cloned in pGBKT7 vector to give clones pGBK-PvTRAg36.6 and pGBK-PvTRAg56.2. The PvMSP7 and PvETRAMP genes were amplified (using primer sets, 5’–*GGATCC*ATATGAAGGGCCGAATCGTGC—3’ and 5’–*CTCGAG*CTAAAGCTCAAGGGTGTTC– 3’ for PvMSP7, and 5’–*GGATCC*ATATGAAAATTACCAAAGTATTATACCT– 3’ and 5’—*CTCGAG*TTAT TGGATGTTGCTGCCTTTGGTTG– 3’ for PvETRAMP, respectively) and cloned into pGAD vector to generate clones pGAD-PvMSP7and pGAD-PvETRAMP. Interaction of these proteins in yeast was assessed by co-transformation and selection on dropout media as described earlier [29].

### Co-immunoprecipitation and protein identification by mass spectrometry

Four hundred μl of trophozoite and schizont stage infected RBCs obtained after layering on 60% percoll, were incubated with 4 hemolytic units of streptolysin O (Sigma-Aldrich, San Louis, MO, USA) in 1x-PBS at room temperature for 10 min and centrifuged at 10,000 x g for 15 sec and washed twice in 1x-PBS. Co-immuno precipitation was done using anti-GFP antibody (Sigma-Aldrich) and Crosslink-IP kit (Thermo Fisher Scientific Inc., Rockford, USA) following manufacturer’s instructions. Direct analysis of immunoprecipitated eluates, in solution digestion and analysis was done as described previously [30].

### Recombinant PvTRAg expression and generation of immune sera

The exon 2 of PvTRAgs36.6 and PvTRAg56.2 were subcloned into the pET28a and pET32a (Invitrogen Life Technologies), respectively, for protein expression in *E*.*coli*. Expression and purification of the recombinant histidine-tagged PvTRAg36.6 and thioredoxin-tagged PvTRAg56.2 was carried out using the same method as described earlier [31]. Proteins were checked on 12% SDS-PAGE and confirmed by western blot analysis using monoclonal anti-His_6_ antibody (Sigma-Aldrich).

Protein specific polyclonal antibodies were raised in rabbit and mice as described earlier [32]. Briefly, New Zealand White rabbits were immunized with purified recombinant protein. Approximately 300 μg of the fusion protein was emulsified with equal volume of Complete Fruend’s Adjuvant and injected intra-dermally at multiple sites. After three weeks of primary immunization, three consecutive boosters were given subcutaneously at two weeks’ intervals with approximately 200 μg of the fusion protein emulsified in incomplete Fruend’s Adjuvant. Ten days after the last immunization, blood was collected and the separated serum was stored at -20^0^ C. To raise antibodies in BALB/c mice, 50 μg of the fusion protein emulsified with Complete Fruend’s Adjuvant was injected intraperitoneally. Mice were administered with two booster doses (25 μg of protein) on day 14 and day 28 formulated in incomplete Freund’s adjuvant. The final bleed was collected 10–14 days after the third boost. A pre-immune serum was collected from the animals prior to first immunization. For blood withdrawal, rabbits were bled through marginal ear vein while mice through the lateral tail venipuncture without sacrificing the animals. Animals were housed and maintained at 22^0^ C—24°C with 50–60% humidity, 300–350 Lux with regular 12 h light cycle, and 10–15 air changes per hour ventilation at the Institute’s Central Animal Facility. Mice were fed with Pellet diet of 5–8 g/day with water intake of 3–7 ml/day. Rabbits were fed with 60 g pellet diet and 100 g of green vegetable (cucumber, carrot, cabbage, etc) diet per day.

### Immunoflourescence microscopy

Immunofluorescence assay was carried out using thin smears of transgenic *P*.*falciparum* culture or from *P*.*vivax* infected patients blood as described earlier [32] using primary antibody [rabbit anti-PvTRAg36.6 (1:100), mice anti-Band3 (1:600), mice anti-PvTRAg36.6 (1:100), rabbit anti-PfClpP (1:100), rabbit anti-PvRII (1:200), mice anti-PvAARP (1:100), mice anti-PvTRAg56.2 (1:500), rabbit anti-PvMSP1-19 (1:200), rat anti-PfSBP1(1:200), rabbit anti-GFP (1:700), mice anti-PfSERA5 (1:200), mice anti-spectrin (1:600), mice anti-GFP (1:700)] and secondary antibody [anti-mice Alexafluor 488 (1:700), anti-rabbit Alexafluor 594 (1:700), anti-mice Alexafluor 594 (1:700), anti-rabbit Alexafluor 488 (1:700), anti-rat, Alexafluor 594 (1:700)] diluted in 1% fetal calf serum (FCS). Smears were mounted using Fluoroshield (Sigma-Aldrich) containing 4', 6-diamidino-2-phenylindole (DAPI). Images were obtained under 100 x immersion oil objective lens using Nikon Eclipse E600 fluorescent microscope or Nikon confocal microscope A1 (Nikon Corporation, Tokyo, Japan).

### Plasmid constructs and *P*. *falciparum* parasite transfection

Entire open reading frame of PvTRAg36.6 (960 bp) and PvTRAg56.2 (1394 bp) genes were custom synthesized commercially (Biolinkk, New Delhi, India) and were cloned into transfection vector pARL1a-GFP which expresses genes under the control of *Pfcrt* (*P*.*falciparum* chloroquine resistant transporter) promoter, in *KpnI* and *BglII* cloning sites to generate transgenic constructs: pARL1a-PvTRAg36.6 and pARL1a PvTRAg56.2. The PvTRAg56.2 gene was also cloned in pARL1a-GFP vector consisting of late stage promoter, *pfama1* (*P*.*falciparum* apical membrane antigen).

*Plasmodium falciparum* 3D7 parasites were cultured with human erythrocytes at 4% haematocrit in RPMI media (Gibco Co, Auckland, Grand Island, USA) supplemented with 5% Albumax I (Gibco) following Trager and Jensen (Trager and Jensen, 1976). Synchronized ring stage parasites were transfected with 100 μg of each recombinant plasmid [33] by electroporation (310V, 950 μF). Parasites were maintained under the 5 nM WR99210 drug pressure. Resistant parasites appeared after one to two months of transfection.

To ascertain the expression of PvTRAg-GFP fusion proteins in the transgenic line, parasite lysates were subjected to 10% SDS-PAGE and western blot analyses using monoclonal anti-GFP antibody (1:2000 dilution).

## Results

### Construction of pTRG-*P*.*vivax* cDNA asexual blood stage library

We have constructed a cDNA library from total RNA pooled from 12 *P*.*vivax* infected human patients using bacterial two hybrid target vector. Percentage of recombinant clones was more than 80% with library titer of 5×10^6^ cfu/ml. DNA sequencing of randomly picked up clones revealed that genes expressed during all blood stages, including gametocytes, were represented in the library. However, high percentage of clones encoding hemoglobin was represented in the library. The *P*.*vivax* cDNA library was subsequently screened against PvTRAg36.6 and PvTRAg56.2.

### Identification of PvTRAg36.6 and PvTRAg56.2 interacting proteins

Plasmids isolated from 85 and 92 positive clones for PvTRAg36.6 and PvTRAg56.2, respectively, were sequenced. BLAST search of the DNA sequences at NCBI (http://www.ncbi.nlm.nih.gov/pubmed) as well as PlasmoDB (http://plasmodb.org/plasmo/) database identified several interacting proteins for each PvTRAg (**Table 1**). Majority of the clones showed the sequence of abundantly expressed genes. Getting rid of abundantly expressed proteins becomes difficult even after increasing the stringency of experiments to the highest level. For this very reason Pvs25 along with other abundantly expressed proteins such as histones and transcription factors were considered as false positive and negated. Therefore, after this scrutinization, PvETRAMP and PvMSP7 were considered as putative interacting partners for PvTRAg36.6 and PvTRAg56.2, respectively (**Table 1**).

**Table 1:** Putative interacting protein partners of PvTRAg36.6 and PvTRAg56.2 identified by bacterial two hybrid assay.

Protein name(Accession no.)	Putative interacting protein	Accession no.	Function/localization
**PvTRAg36.6(PVX_112690)**			
	Ookinete surface protein Pvs25	PVX_111175	Oocyst development/gametocyte surface
	Early transcribed membrane protein (ETRAMP)	PVX_003565	Unknown/PVM ^a^, MC ^b^
	Hypothetical protein beta-hydroxyacyl-ACP	PVX_116720	Fatty acid synthesis / Apicoplast
	Histone H3, putative	PVX_114020	chromatin associated/Nucleus
	Replication factor a protein, putative	PVX_000765	DNA replication/Nucleus
	Hypothetical protein, conserved	PVX_113580	Unknown
**PvTRAg56.2(PVX_088850)**			
	Merozoite surface protein 7 (MSP7)	PVX_082645	Associates with MSP1 / merozoite surface
	Hypothetical protein	PVX_114665	Transcription factor Tfb4 domain /nucleus
	Ookinete surface protein Pvs25	PVX_111175	Oocyst development /gametocyte surface
	Variant surface proteins Vir22/5/24, putative	PVX_119210	Variant surface antigen/Infected RBC membrane
	Hypothetical protein	PVX_003940	Ring domain, ubiquitination
	Hypothetical protein	PVX_095205	Unknown
	Conserved protein	PVX_089515	DUF1777domain, unknown function
	Elongation factor alpha	PVX_114830	Translation/Cytoplasm
	Basic transcription factor 3b	PVX_085220	Transcription/Nucleus

^a^ PVM-Parasitophorous vacuole membrane

^b^ MC-maurer’s cleft.

To further confirm the specificity of the identified interactions, we performed yeast two hybrid analysis. For this, pGBK-PvTRAg36.6 and pGBK-PvTRAg56.2 recombinant plasmids were co-transformed with pGAD-PvETRAMP and pGAD-PvMSP7, respectively, into the AH109 yeast cells. Potential interacts were assessed by growth of the co-transformants on selective media. Yeast cells co-transformed with pGBK-AL4 and pGAD-AL1 (positive control); pGBK-PvTRAg56.2 and pGAD-PvMSP7; pGBK-PvTRAg36.6 and pGAD-PvETRAMP were able to rescue, while, pGBK-PvTRAg36.6 and pGBK-PvTRAg56.2 co-transformed with empty pGADT7 vector as control, did not grow on the selective media **(S1 Table)**. Results confirmed PvETRAMP and PvMSP7 as interacting partners of PvTRAg36.6 and PvTRAg56.2, respectively.

**Fig 1:**
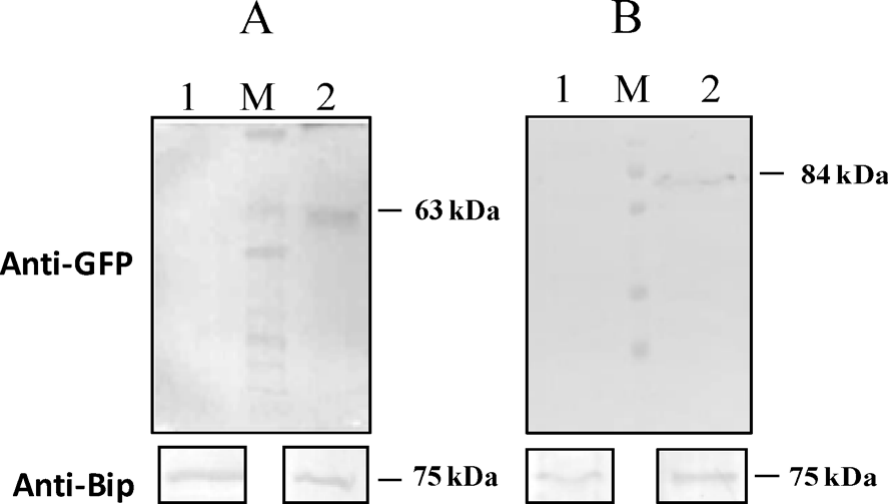
Expression of PvTRAg-GFP fusion proteins in transgenic *P*.*falciparum*. Parasite lysates from wild type 3D7, transgenics 3D7_PvTRAg36.6-GFP and 3D7_PvTRAg56.2-GFP were subjected to western blot analyses using monoclonal anti-GFP antibody (upper panels) and anti-Bip antibody as control (lower panels). A: lane 1; wild type 3D7; Lane 2; 3D7_PvTRAg36.6-GFP, B: lane 1; wild type 3D7; lane 2; 3D7_PvTRAg56.2-GFP, Molecular weights of GFP fusion protein and parasite Bip protein are indicated. M shows the protein marker.

To identify the proteins that interact with PvTRAg under native conditions, we expressed respective PvTRAg trans-gene fused with GFP in *P*.*falciparum*. Expression of GFP fusion protein was confirmed by western blot analysis where the protein bands of expected sizes were detected from parasite lysates probed with anti-GFP antibody **(Fig 1).** No corresponding protein bands were observed for wild type *P*.*falciparum* 3D7 parasite lysate (negative control). Polyclonal anti-BiP antibody detected abundantly expressed ER protein BiP in wild type as well as transgenic parasite (Positive control). Proteomics analysis of immunoprecipitates from these transgenic parasites lysates by anti GFP antibody identified several proteins which directly or transiently interact during protein trafficking **(S2 Table)**. PvTRAg36.6 seems to interact with PVM localized ETRAMP4 and proteins which are known to be trafficked to the red cell membrane via Maurer’s clefts including PfEMP, Pf332, as well as rhoptry proteins including RON4, CLAG9 while PvTRAg56.2 interacted with Merozoite surface protein 9 (MSP9), Glycophorin binding protein (GBP), Merozoite surface protein 7 (MSP7). Identification of ETRAMP4 and MSP7 as an interacting partner of PvTRAg36.6 and PvTRAg56.2, respectively, was in corroboration to the bacterial two hybrid results **(Table 1)**.

### PvTRAg36.6 is apically localized in merozoites

**Fig 2:**
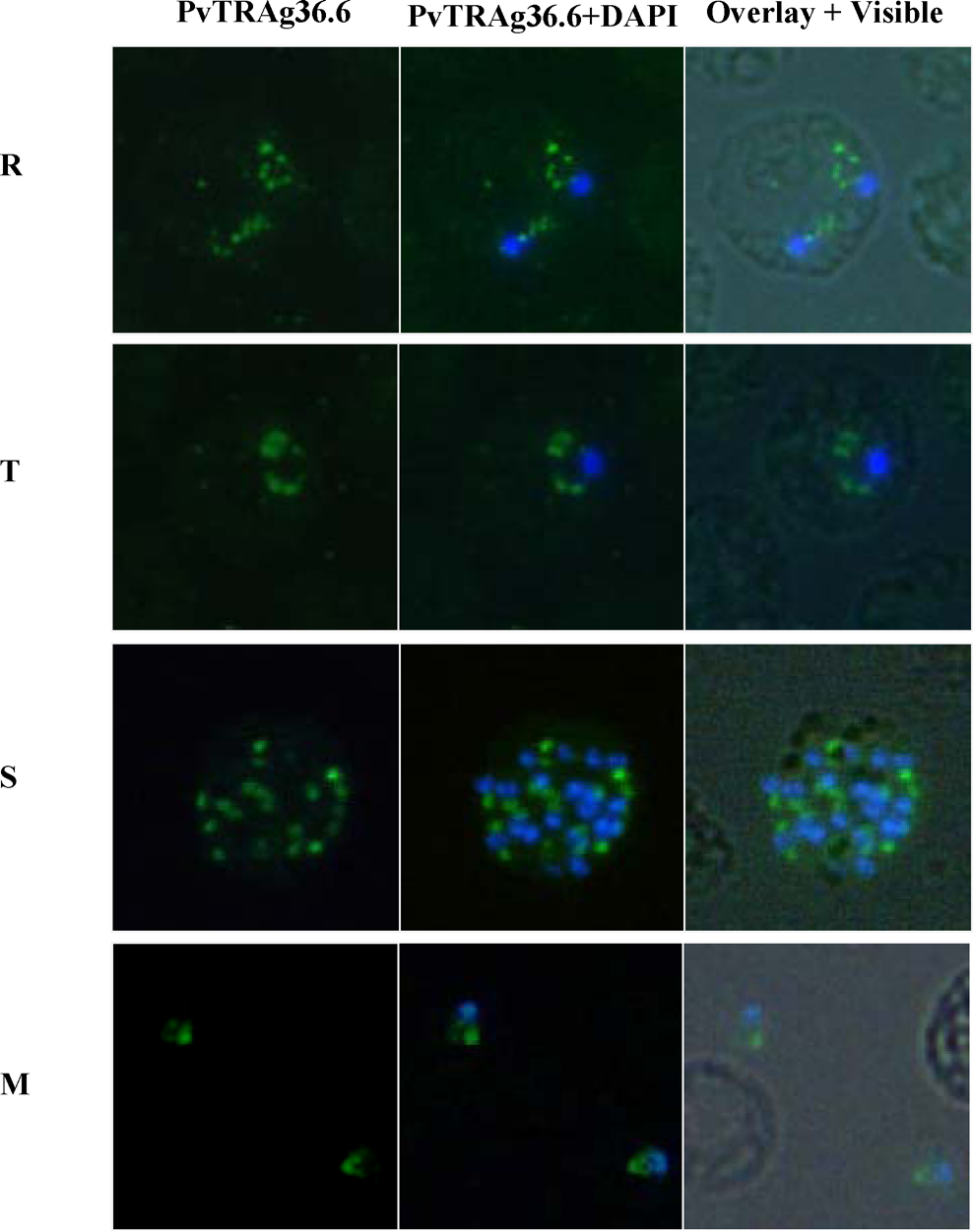
Sub cellular localization of PvTRAg36.6 in *P*.*vivax* natural infections. Immunofluorescence images of *P*.*vivax* infected red cells. Parasites were labeled with anti- PvTRAg36.6 (green) antibody and DAPI for nuclear staining (blue). Fluorescence pattern observed in ring (**R**, double infection), trophozoite **(T),** Schizont **(S)** stages and in free merozoites **(M)** are shown. Overlay shows the images merged with bright field.

**Fig 3:**
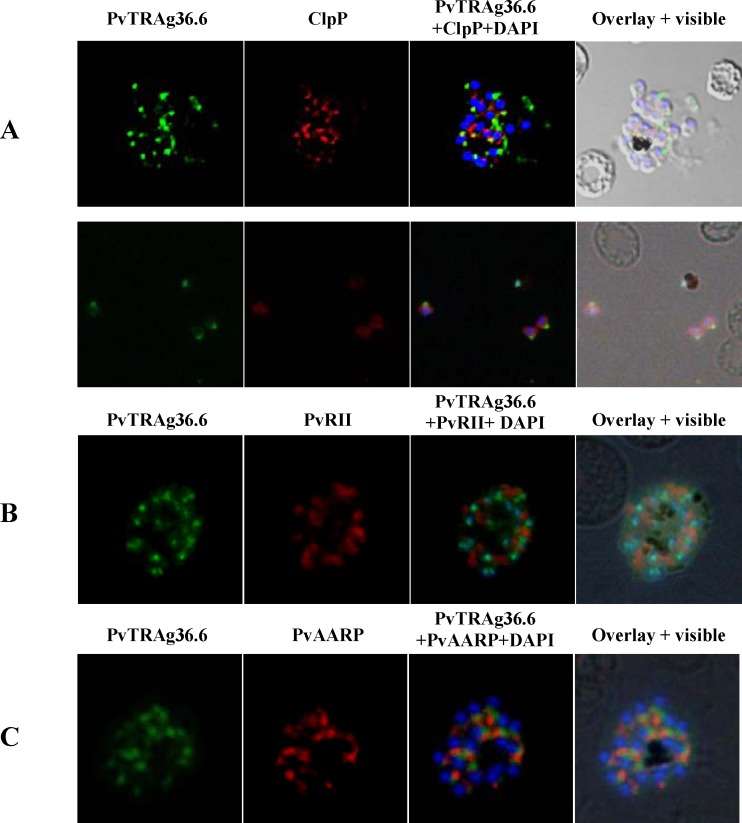
Co-localization studies of PvTRAg36.6 in *P*.*vivax*. Co-localization images of PvTRAg36.6 with apicoplast, rhoptry and micronemal markers in *P*.*vivax* natural infections. **(A)** Fluorescence pattern observed after co-immuno staining of *P*.*vivax* parasite with anti-PvTRAg36.6 (green) and anti-PfClpP (red) recognizing apicoplast in schizont (**A**, upper panel) as well as in free merozites (**A**, lower panel). **(B)** Co-immunostaining of anti-PvTRAg36.6 (green) with anti-PvRII (red) recognizing microneme in a schizont, and **(C)** Co-immunostaining of anti-PvTRAg36.6 (green) with anti-PvAARP (red) recognizing rhoptry neck in a schizont. The parasite nuclei were stained with DAPI (blue). Overlay shows the images merged with bright field.

Immunostaining of *P*. *vivax* infected erythrocytes showed PvTRAg36.6 as small discrete foci close to the parasite periphery in ring and trophozoite stages **(Fig 2)**. In schizonts, fluorescence was observed in a punctate manner towards the individual merozite, whereas in free merozoites the staining was restricted to the apical end **(Fig 2)**. Since in-silico analyses predicted PvTRAg36.6 protein to be apicoplast targeted, co-immunostaining was carried out using antibodies against apicoplast protein marker PfClpP [34] and PvTRAg36.6. Results of dual labeling did not show any overlap **(Fig 3A)**. Fluorescence restricted to one end in free merozoites **(Fig 2)** suggested the apical organelle localization of PvTRAg36.6. To confirm this, we used antibodies against apical organelle resident proteins: microneme marker protein PvRII- a *Plasmodium vivax* Duffy binding protein region II, and PvAARP- Apical Asparagine Rich Protein whose homolog in *P*.*falciparum* has been localized in rhoptry neck [32]. However, none of these marker proteins co-localized with PvTRAg36.6 **(****Fig 3B** and 3**C**). Together, these localization studies suggested that apically localized PvTRAg36.6 may not be present in miconeme, rhoptry neck or apicoplast. Overall, the protein seems to be present towards the apical ends of the merozoites.

### PvTRAg56.2 is localized on merozoite surface

**Fig 4:**
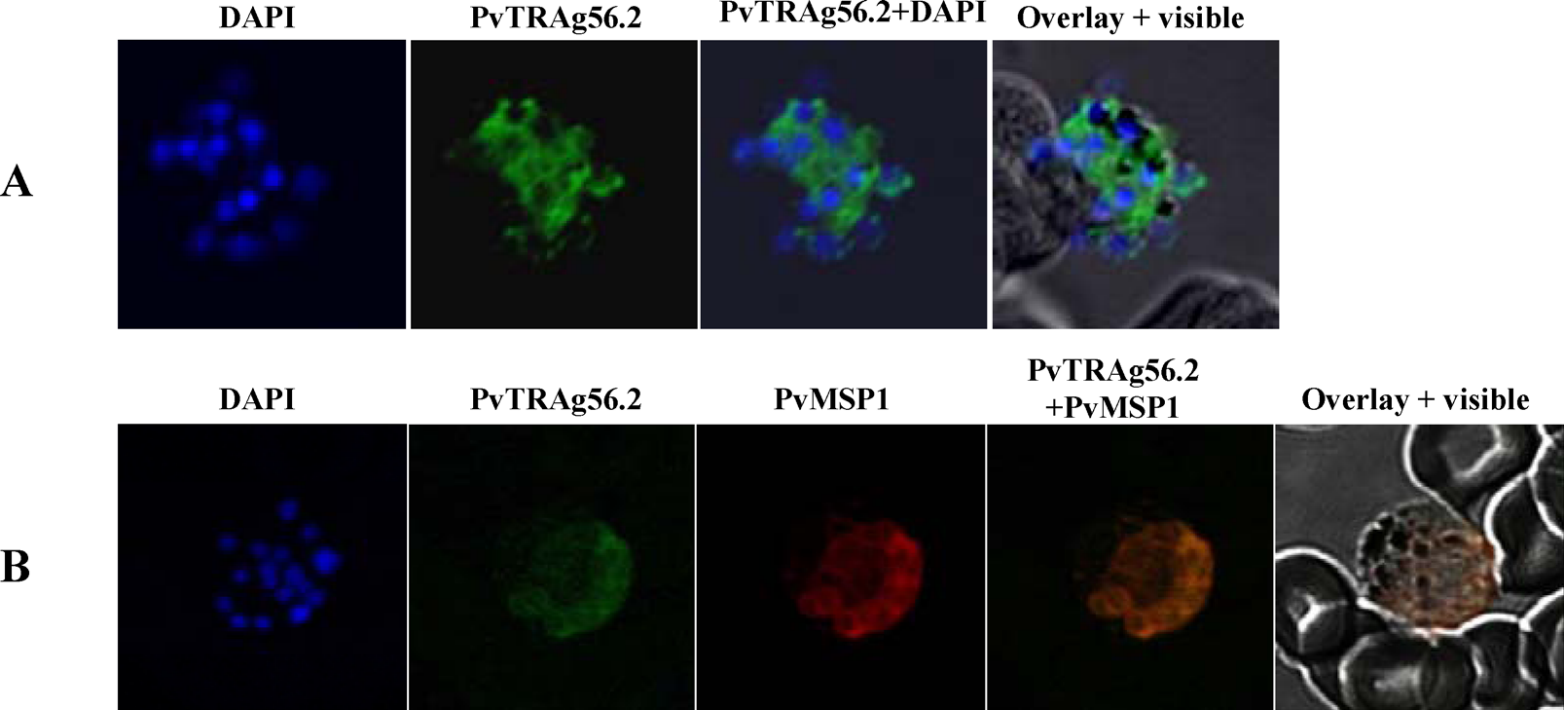
Sub cellular localization of PvTRAg56.2 in *P*.*vivax* natural infections. Localization images of PvTRAg56.2 by immuno and co-immuno staining in *P*.*vivax* natural infections. **(A)** Immunofluorescence pattern observed in *P*.*vivax* schizont when labeled with anti-PvTRAg56.2 (green). **(B)** Co-immunostaining of *P*.*vivax* schizont with anti-PvTRAg56.2 (green) and PvMSP-1(red). Parasite nuclei were stained with DAPI (blue).

PvTRAg56.2 was predicted to be nuclear targeted but immunofluorescence assay with mice anti-PvTRAg56.2 polyclonal antibody against recombinant purified protein clearly localized this protein on merozoites surface **(Fig 4A)**; whereas no staining was observed in rings and trophozoite stage parasites. Further, co-localization was carried out using antibody against *P*.*vivax* merozoite surface protein1 (PvMSP1). The PvTRAg56.2 showed similar labeling as that of PvMSP1 and overlay images confirmed the localization of PvTRAg56.2 on merozoites surface **(Fig 4B)**.

### PvTRAg36.6 and PvTRAg56.2 are exported to the RBC membrane in *P*. *falciparum* transgenic lines

**Fig 5:**
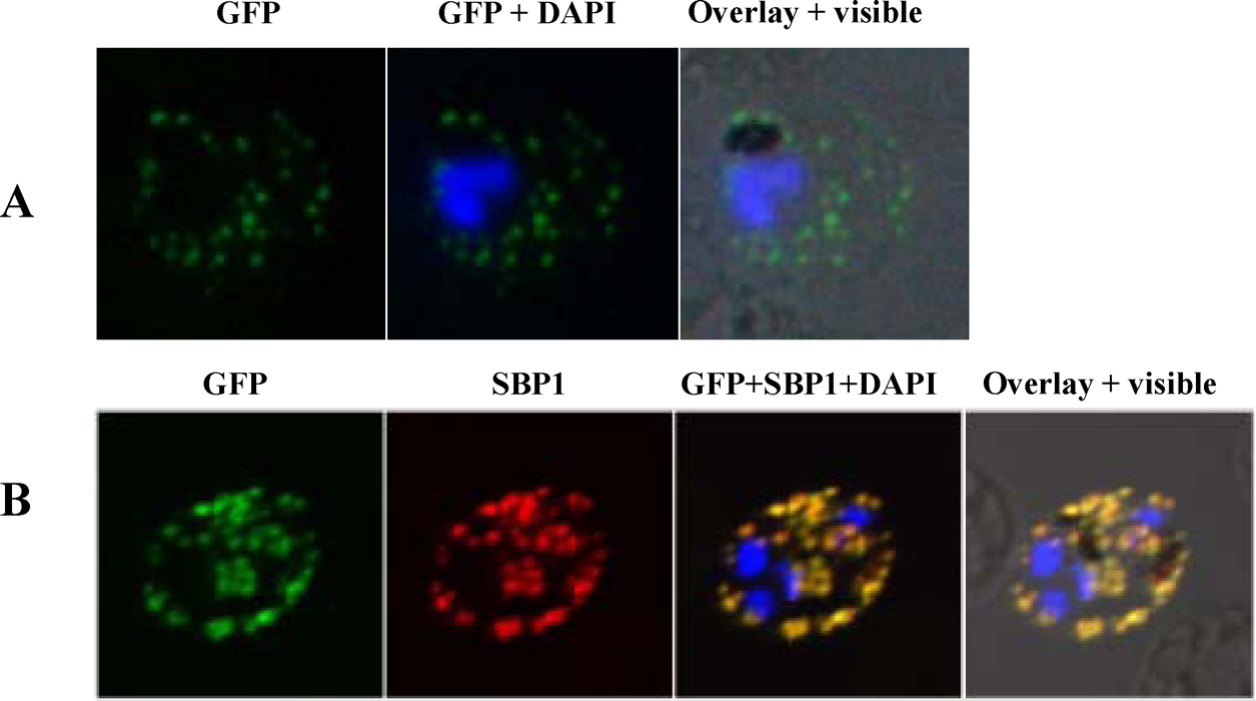
Sub cellular localization of PvTRAg36.6 in *P*.*falciparum* transgenic parasites expressing GFP fusion protein. GFP fluorescence images showing localization of PvTRAg36.6-GFP in trophozoite stages of transgenic parasite line 3D7_ PvTRAg36.6-GFP, B. Images of co-immunostaining between anti-GFP antibody (green) and anti-SBP1 antibody (red). Parasite nuclei were labeled with DAPI (blue). Overlay shows images merged with bright field.

To confirm the localization, the transgenic *P*.*falciparum* lines expressing PvTRAg36.6-GFP and PvTRAg56.2-GFP were used. PvTRAg36.6 in transgenic *P*.*falciparum* was present as punctuate structure around the parasite in trophozoite stages, suggesting that it is localized in PV **(Fig 5A).** Further, the GFP labeling was also found to be present in cytosol of infected RBC as flattened disc like structures **(Fig 5B)**. RBC membrane associated staining was also seen as the parasite developed. To further confirm the sub cellular location of PvTRAg36.6-GFP, immunostaining was carried out for the Maurer’s cleft resident protein PfSBP1 (*P*.*falciparum* Skeletal binding protein1) using anti-PfSBP1 antibody. Results showed complete co-localization between the PvTRAg36.6-GFP and integral membrane protein PfSBP1 **(Fig 5B)**, confirming the export of PvTRAg36.6-GFP to the RBC membrane via Maurer’s clefts.

**Fig 6:**
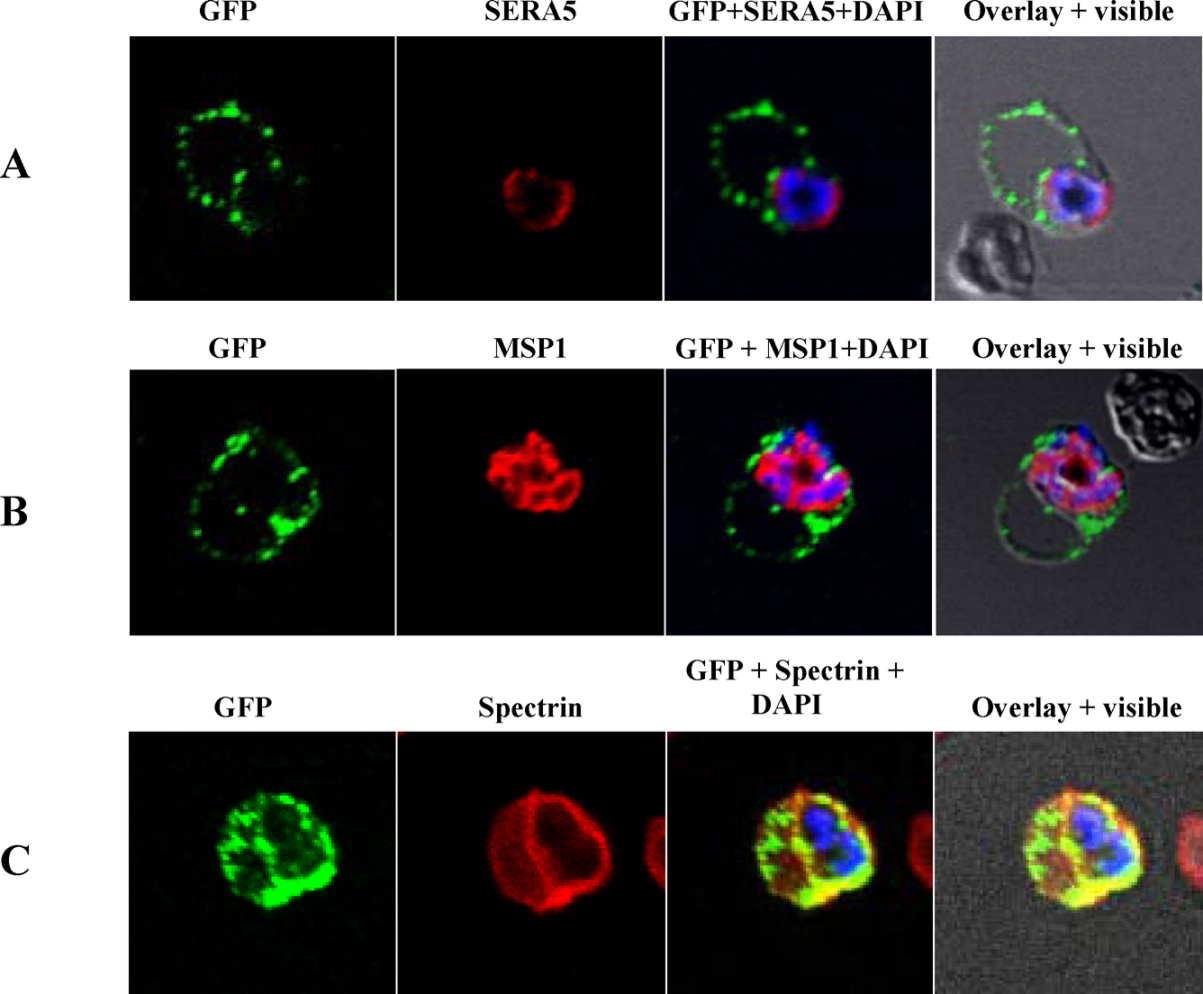
Sub cellular localization of PvTRAg56.2 in *P*.*falciparum* transgenic parasites expressing GFP fusion protein. Images of co-immunostaining between anti-GFP (green) with (A) anti-SERA5 (red) or (B) anti-PfMSP-1 (red) or (C) anti-Spectrin (red) in transgenic parasite line 3D7_ PvTRAg56.2-GFP. Parasite nuclei were labeled with DAPI (blue). Overlay with bright field images are shown.

In transgenic line expressing PvTRAg56.2-GFP fusion protein, fluorescence was seen as distinct dots around the parasite periphery and as non uniform fluorescence speckles at the infected RBC membrane as well **(Fig 6)**. To further study the localization pattern in *P*.*falciparum*, immunostaining studies were carried out for PfSERA5, a soluble parasitophorous vacuole (PV) resident protein, and Spectrin, which is a red cell cytoskeleton protein present at the intracellular face of RBC membrane. Co-localization of PvTRAg56.2-GFP with PfMSP-1 (as seen above in natural infection) was done to look for any direct or transient interactions between them during trafficking. Results of these immunostaining studies showed no co-localization of PvTRAg56.2-GFP with PfSERA5 **(Fig 6A)** or PfMSP1 **(Fig 6B)**. PvTRAg56.2-GFP staining did not overlap but was found to be closely associated with the SERA5 staining suggesting protein to be PVM associated rather than as a soluble protein in PV. Regions of dual staining with Spectrin confirmed PvTRAg56.2 to be RBC membrane associated **(Fig 6C)**. Together, results showed that PvTRAg56.2 is associated with parasite plasma membrane and also gets exported to the RBC membrane following the different trafficking route when compared to PvTRAg36.6.

## Discussion

Comparative genomic studies have shown that *P*.*vivax* encodes for many more members of a ‘Pvfam-a’, multigene family in comparison to *P*. *falciparum* that encodes only five members [2]. Our group has been working on functional characterization of many of these proteins and has characterized their antigenicity in patient sera [10, 28]. We have also illustrated that some of these proteins bind RBC and proposed that these may have a vaccine potential. However, question still remains that why *P*.*vivax* encodes for such a large number of tryptophan-rich proteins and how does these proteins differ in their function(s)? It has been difficult to characterize *P*. *vivax* proteins because of lack of an *in vitro* culture system and most of the work has to be performed in *P*. *vivax* parasites isolated from patient blood samples. To understand the functional diversity among members of ‘Pvfam-a’ multigene family in the present study, we characterized two members of the family, PvTRAg36.6 and PvTRAg56.2, for their interacting partners and by sub-cellular localization studies.

Since it is difficult to carry-out gene knock-out studies in *P*. *vivax* parasites for functional genomic studies, to get insight into the role(s) of PvTRAg36.6 and PvTRAg56.2 antigens we generated a *P*. *vivax* cDNA expression library and screened these two antigens for their interacting partners. In addition, we also generated *P*. *falciparum* transgenic lines expressing PvTRAg36.6-GFP and PvTRAg56.2-GFP fusion proteins. Among the different proteins PvETRAMP and PvMSP7 were identified as interacting partner for PvTRAg36.6 and PvTRAg56.2 respectively, after bacterial two hybrid screening. Same two antigens were identified by immunoprecipitation analysis of transgenic *P*. *falciparum* parasites expressing PvTRAg36.6-GFP and PvTRAg56.2-GFP fusion proteins using anti-GFP antibody followed by LC-MS/MS **(S2 Table)**. We further confirmed the specificity of PvTRAg36.6 interaction with PvETRAMP and, PvTRAg56.2 interaction with PvMSP-7 using yeast two hybrid analyses **(S1 Table)**. The immunofluorescence assay showed that PvTRAg36.6 was apically localized in free *P*.*vivax* merozoites. It was also seen in non-uniform discrete dotted pattern at the parasite periphery during ring as well as trophozoite stages, and as vesicular structures near each nucleus in schizont stage. These results are in line with the protein-protein interaction data that showed PvETRAMP as its interacting partner as, ETRAMP family members have been characterized as the most prominent group of parasitophorous vacuolar membrane (PVM) resident proteins and have been implicated as important mediators of host-cell interface interaction [35, 36]. Parasite invades the red cell and resides in parasitophorous vacuole (PV) [37–39] enclosed by PVM which forms the interface between parasite and its host. In *P*.*falciparum* as well as in a related parasite *Toxoplasma gondii*, apical organelle secreted parasite proteins are targeted to the PVM/PV and contribute to its development. Proteins such as Rhoptry associated membrane antigen (RAMA) [40, 41], high molecular weight (HMW) rhoptry complex [42], low molecular weight (LMW) complex and Stomatin in *P*.*falciparum* have been implicated in this process [43]. *Plasmodium falciparum* dense granules secreted proteins like Ring exported surface antigen (RESA) and Ring membrane antigen (RIMA) are shown to be localized in PVM and implicated in establishment of PV [44, 45]. Like-wise rhoptry proteins; ROP1, 2, 3, 4, 7 and ROP5 of *Toxoplasma gondii* are also targeted to the PVM [46] in a process of host cell invasion and PVM formation. Therefore, it is possible that apically localized PvTRAg36.6 is secreted at some point of time during invasion and targeted to the parasite periphery in ring and trophozoite stages to play role in PVM development or maintenance. It is known in certain cases that proteins targeted to apical organelles can appear much before the organelles themselves [40], this could be the case with PvTRAg36.6 which is transcribed in late ring/trophozoite but is also observed in schizonts/merozoites stages of the parasite [14]. It is possible that due to its apical localization as well as at the time of secretion; the protein gets exposed to the immune system. This is evident from our previous study that showed RBC binding activity for PvTRAg36.6 antigen [16] and immune response against this protein in *P*.*vivax* natural infections [10].

In comparison to PvTRAg36.6 localization, late stage transcribed PvTRAg56.2 [14] was found surrounding the merozoites in schizonts by immunofluorescence localization in *P*.*vivax*. This localization pattern was similar to that of the merozoite surface proteins especially the PvMSP1 in natural infection. Merozoite surface protein 7 (MSP7) was identified as molecular interacting partner of PvTRAg56.2 by bacterial two hybrid system which was further confirmed by yeast two hybrid assay. The surface of the merozoite is composed of many integral and peripheral membrane proteins that have been termed as merozoite surface proteins (MSPs), these proteins are important components in the erythrocyte invasion mediating the relatively weak and reversible initial interactions between the parasite and RBC [47]. In *P*.*falciparum*, PfMSP7 is a part of protein complex present on the merozoite surface in association with PfMSP1, PfMSP6 and some other proteins [48–51]. But in *P*.*vivax* not all counterparts of this complex are found [2], however MSP7 protein coding family is well expanded in *P*.*vivax*, with eleven genes as compared to six members in *P*.*falciparum*. It is not known if any of the *P*.*vivax* MSP7 proteins bind to PvMSP1 protein as observed in *P*.*falciparum*. Since recombinant protein PvTRAg56.2 does not show erythrocyte binding activity [16] it is likely that, PvTRAg56.2 and PvMSP7 interact together to assist the establishment or stabilization of protein complex at *P*.*vivax* merozoite surface, similar to MSP1 complex observed in *P*.*falciparum*. However, further assessment of PvMSP1 interaction with PvTRAg56.2 and PvMSP7 can provide significant insights. It may be noted here that during cellular processes a protein interacts with array of other protein partners either stably or transiently, therefore identification of ETRAMP and MSP7 highlights only one of the many possible interacting partners of PvTRAgs. Further investigations with uncharacterized hypothetical proteins identified by bacterial two hybrid **(Table 1)** or immunoprecipitation assay **(S2 Table)** can help to identify more such protein partners.

To better understand the localization and trafficking of PvTRAg36.6 and PvTRAg56.2 proteins, we made detailed localization studies in *P*. *falciparum* transgenic lines expressing PvTRAg36.6-GFP and PvTRAg56.2-GFP proteins. We observed that PvTRAg36.6 and PvTRAg56.2 were exported to the RBC membrane **(Figs 5 and 6)** following different trafficking routes. However, differences were observed in localization data by fluorescence assays and transgenic lines expressing GFP fusion protein. This is probably due to different ways the protein signals are recognized by sorting machinery in these two parasite species. It is possible that in heterologous system due to unavailability of proteins, which may assist correct targeting, PvTRAgs were mistargeted to RBC membrane. This can be exemplified by the mistargeting of RAP 2 protein to PV rather than the rhoptries in absence of RAP 1 protein [52]. Also, mistargeting of PvTRAg36.6 and PvTRAg56.2 in absence of native promoter or UTRs cannot be ruled out.

In conclusion, identification of different interacting cellular partners for two PvTRAgs and their differential localization suggested that tryptophan rich proteins in *P*.*vivax* play differential role(s) in parasite survival and growth. Further studies on these proteins may be useful to develop new *P*. *vivax* specific anti-malarial strategies.

## Supporting Information

S1 Table Growth phenotype of yeast AH109 cells co-transformed with different PvTRAg constructs. (PDF)

S2 Table Interacting protein partners of PvTRAg36.6 and PvTRAg56.2 as identified by Pull down assay from 3D7 transgenic line. (PDF)
