# Supplementary material for: Plasmodium vivax Tryptophan Rich Antigen PvTRAg36.6 Interacts with PvETRAMP and PvTRAg56.6 Interacts with PvMSP7 during Erythrocytic Stages of the Parasite
Source: PLoS One. 2016 Mar 8;11(3):e0151065. doi: 10.1371/journal.pone.0151065 (PMC4783080; doi:10.1371/journal.pone.0151065)
Supplement: S1 Table — (PDF) [file pone.0151065.s001.pdf]

Table S1. Growth phenotype of yeast AH109 cells co-transformed with different PvTRAg constructs

S.No.	Transformation constructs		Selection on dropout media		
	BAIT	TARGET	SD-Leu$^-$/Trp$^-$	SD-Leu/Trp/His$^-$	SD-Leu/Trp/His/Ade$^-$
1	-	pGAD vector	-	-	-
2	pGBK vector	-	-	-	-
3	pGBK-AL4	pGAD-AL1	+	+	+
4	pGBK-PvTRAg36.6	pGAD vector	+	-	_
5	pGBK-PvTRAg36.6	pGAD-PvETRAMP	+	+	+
6	pGBK-PvTRAg52.6	pGAD vector	+	-	-
7	pGBK-PvTRAg52.6	pGAD-PvMSP7	+	+	+

SD-Leu$^-$/Trp, leucine and tryptophan double dropout; SD-Leu/Trp/His, , leucine, tryptophan and histidine triple dropout; SD-Leu/Trp/His/Ade$^-$, leucine, tryptophan, histidine and adenine quadruple dropout.
