# Supplementary material for: Plasmodium vivax Tryptophan Rich Antigen PvTRAg36.6 Interacts with PvETRAMP and PvTRAg56.6 Interacts with PvMSP7 during Erythrocytic Stages of the Parasite
Source: PLoS One. 2016 Mar 8;11(3):e0151065. doi: 10.1371/journal.pone.0151065 (PMC4783080; doi:10.1371/journal.pone.0151065)
Supplement: S2 Table — (PDF) [file pone.0151065.s002.pdf]

Table S2. Interacting protein partners of PvTRAg36.6 and PvTRAg56.2 as identified by Pull down assay from 3D7 transgenic line*

	Interacting protein	GenBank ID	No. of peptides identified	Molecular Weight (kDa)	Sequence coverage (%)
**PvTRAg36.6**	Circumsporozoite-related antigen, exported protein 1 (EXP1)	PF3D7_1121600	4	17.2	36.42
	Early transcribed membrane protein 4 (ETRAMP4)	PF3D7_0423700	2	14.8	28.68
	Cytoadherence linked asexual protein 9 (CLAG9)	PF3D7_0935800	3	160.3	3.28
	Plasmodium exported protein, unknown function	PF3D7_0701900	1	112.6	2.07
	Plasmodium exported protein, unknown function	PF3D7_1353100	3	32.7	12.63
	Plasmodium exported protein, unknown function	PF3D7_0730900	1	244.0	1.71
	Duffy binding-like merozoite surface protein (DBLMSP)	PF3D7_1035700	1	80.1	1.17
	Rhoptry neck protein 4 (RON4)	PF3D7_1116000	1	135.4	1.17
	Antigen 332, DBL-like protein (Pf332)	PF3D7_1149000	1	688.8	0.11
	Erythrocyte membrane protein 1, PfEMP1 (VAR)	PF3D7_1240300	1	300.0	2.04
**PvTRAg56.2**	14-3-3 protein (14-3-3I)	PF3D7_0818200	8	30.2	41.2
	Merozoite surface protein 9 (MSP9)	PF3D7_1228600	9	86.5	21.8
	Rhoptry-associated protein 1 (RAP1)	PF3D7_1410400	9	90.0	20.3
	Enolase (ENO)	PF3D7_1015900	5	48.6	23.5
	Actin I (ACT1)	PF3D7_1246200	5	41.8	20.7
	Glycophorin binding protein (GBP)	PF3D7_1016300	4	95.8	30.7
	High molecular weight rhoptry protein 3 (RhopH3)	PF3D7_0905400	3	104.8	6.24
	Merozoite surface protein 7 (MSP7)	PF3D7_1335100	2	41.3	7.6
	Serine repeat antigen 5 (SERA5)	PF3D7_0207600	3	111.7	6.0
	Tubulin beta chain	PF3D7_1008700	2	49.7	8.5

*proteins precipitated using anti-GFP antibody, after In-solution trypsin digestion were identified by Liquid chromatography-tandem mass spectrometry (LC-MS/MS).
